# Perinatal folate-related exposures and risk of psychotic symptoms in the ALSPAC birth cohort^☆^

**DOI:** 10.1016/j.schres.2010.03.006

**Published:** 2010-07

**Authors:** B. Glaser, A.E. Ades, S. Lewis, P. Emmet, G. Lewis, G. Davey Smith, S. Zammit

**Affiliations:** a The MRC Centre for Causal Analyses in Translational Epidemiology, University of Bristol, Oakfield House, Oakfield Grove, Bristol, BS8 2BN, UK; b Department of Social Medicine, University of Bristol, Canynge Hall, 39 Whatley Rd, Bristol BS8 2PS, UK; c Department of Community Based Medicine, Cotham House, University of Bristol, Cotham Hill, Bristol, BS6 6JL, UK; d Department of Psychological Medicine, School of Medicine, Cardiff University, Heath Park, Cardiff CF14 4XN, UK

**Keywords:** PLIKS, ALSPAC, *MTHFR* C677T, Folate, Epigenetic

## Abstract

**Background:**

It is unclear to what extent non-clinical psychotic experiences during childhood and adolescence share underlying aetiological mechanisms with schizophrenia. One candidate mechanism for schizophrenia involves the epigenetic status of the developing fetus, which depends on the internal folate-status of mother and child. Our study examines the relationships between multiple determinants of perinatal folate-status and development of psychotic experiences in adolescence.

**Methods:**

Study participants were up to 5344 mother–child pairs from the Avon Longitudinal Study of Parents and their Children, UK, with information on maternal and/or child *MTHFR* C677T genotype, maternal folate intake (supplementation at 18/32- weeks gestation; dietary intake at 32- weeks gestation) and psychosis-like symptoms (PLIKS) for children assessed at age 12.

**Results:**

Nominal evidence was observed that maternal folate supplementation at 18 weeks increased the odds of PLIKS in children (odds ratio(OR) = 1.34; 95%-CI:[1.00;1.76]) and, consistent with this, that children of *MTHFR* C667T TT homozygous mothers had decreased odds of PLIKS (OR = 0.72; 95%CI:[0.50;1.02]; recessive model) with strongest effects in boys (OR = 0.44, 95%-CI:[0.22;0.79]; sex-specific *p* = 0.029). None of the reported effects remained significant when corrected for multiple testing.

**Conclusions:**

Overall, this study found no support that maternal/child *MTHFR* C677T genotype and maternal folate intake during pregnancy contribute to common aetiological pathways that are shared between schizophrenia and non-clinical psychotic symptoms in adolescents, assuming that decreased folate-status increases schizophrenia risk.

## 1 Introduction

Non-clinical psychotic symptoms on interview, including delusions and hallucinations, have been reported for up to 18% of adults (Eaton et al., 1991; Johns et al., 2004; Scott et al., 2006; van Os et al., 2001; Wiles et al., 2006) and 14% of children based on observer-rated assessment (Poulton et al., 2000). These proportions exceed by far the life-time cumulative incidence of any psychotic condition in the general population, which reaches approximately 2 to 3% (Kendler et al., 1996; Perälä et al., 2007). The mechanisms linking both psychotic experiences and schizophrenia are not yet understood.

Findings from the Dunedin cohort suggest the continuity of psychotic symptoms from childhood to adulthood within a small proportion of children, such that subclinical psychotic experiences precede later development of a psychotic condition (Poulton et al., 2000). This accentuates the possibility that both schizophrenia and non-clinical psychotic symptoms share underlying aetiological mechanisms.

A rising body of evidence suggests epigenetic modification as a plausible mechanism that leads to neuropsychiatric conditions (Bassett et al., 2002) including schizophrenia (Gräff and Mansuy, 2008). DNA methylation is an important epigenetic regulator of gene expression, growth and tissue differentiation (Suzuki and Bird, 2008). It has been hypothesised that aberrant DNA methylation might be implicated within the aetiology of psychotic disorders through abnormal neurodevelopment (Singh et al., 2003) or high-risk conceptions (Jongbloet et al., 2008).

The fidelity of DNA methylation patterns crucially depends on the regulation of the folate- and one-carbon-cycle (McNulty and Scott, 2008). One of the key players in this cycle is methylenetetrahydrofolate reductase (MTHFR). This enzyme reduces 5,10-methylene tetrahydrofolate (THF) irreversibly into 5-methyl THF, the methyl-group donor for the remethylation of homocysteine (Hcy) into methionine (Frosst et al., 1995; Selhub, 2002). A common nonsynonymous *MTHFR* variant has been identified (C677T; rs1801133), which leads to a thermolabile mutant protein with 30% lower enzyme activity *in vitro* (Frosst et al., 1995). The *MTHFR* 677T allele has been linked to increased plasma total Hcy levels (Frosst et al., 1995), which are responsible for the inhibition of the methylation reaction (James et al., 2002). Highest Hcy-levels were observed for TT homozygotes under impaired folate-status (Brattström et al., 1998; Friso et al., 2002).

*MTHFR* C677T variation has been related to a wide spectrum of phenotypes including neural tube disorders (NTD; Blom et al., 2006) and cognition (Roffman et al., 2008). Evidence from recent meta-analyses on schizophrenia suggested that *MTHFR* 677T, in particular the TT genotype (Lewis et al., 2005; Gilbody et al., 2007), confers susceptibility to psychosis, although there appears to be considerable study heterogeneity (Allelic odds ratio (OR): *I*^2^ = 52% to 57%; Allen et al., 2008; Shi et al., 2008).

Within the developing child the internal folate status is also influenced by nutritional exposures, particularly *in utero* and during early postnatal development (Waterland and Michels, 2007), and a deficiency of dietary folate has been associated with impaired DNA methylation (Friso et al., 2002). Low internal folate-status has been suggested as a risk factor for cognitive impairment (Ramos et al., 2005) and appears to contribute to the aetiology of neural tube disorders (NTD; Blom et al., 2006). The latter is of particular interest as NTD and schizophrenia may share underlying risk factors (Zammit et al., 2007) strengthening positions which implicate folate-status within the aetiology of psychosis (Davey Smith and Ebrahim, 2003).

Our study aimed to investigate relationships between non-clinical psychotic symptoms in adolescence and key determinants of fetal folate-status, including maternal folate supplementation and dietary folate intake during pregnancy, as well as maternal and child *MTHFR* C677T genotypes.

## 2 Materials and methods

### 2.1 Sample description

ALSPAC is a population-based prospective birth cohort with extensive data collection on health and development of children and their parents. All pregnant women in the Bristol area (England) with an expected delivery between April 1991 and December 1992 were eligible and approached for participation. 14541 women enrolled within the study and 13988 children were alive at one year. A detailed description of the cohort has been published previously (Golding et al., 2001). Ethical approval was obtained from the ALSPAC Law and Ethics Committee and the Local Research Ethics Committees.

### 2.2 Measurement of folate intake

Three measures of maternal folate intake during pregnancy were investigated in this study as their effects may vary during the course of embryogenesis influencing different aspects of fetal epigenetic re-programming and modification (Dolinoy et al., 2007). Maternal folate supplementation at 18 and 32- weeks gestation was assessed using questionnaire data and based on whether supplements had been taken within the last 3 months. Maternal dietary folate intake (g/week) at 32- weeks gestation, excluding supplements, was measured using food frequency questionnaires (FFQ). For statistical analysis, dietary intake was converted into Z-scores.

Both measures of folate supplementation were correlated with each other (product-moment correlation-coefficient: *ρ* = 0.43; *p* < 0.001) but only marginally related to dietary folate intake (biserial correlation-coefficient: 0.060 < *ρ* ≤ 0.080; 0.016 < *p* ≤ 0.04).

### 2.3 Measurement of psychotic symptoms

Non-clinical psychotic symptoms in children were measured at the age of 12 using the semi-structured psychosis-like symptoms (PLIKS) interview (Horwood et al., 2008) consisting of 12 core questions covering the past 6-month occurrence of hallucinations, delusions and experiences of thought interference. All items followed the glossary definitions of the Schedules for Clinical Assessment in Neuropsychiatry (Wing et al., 1990). Symptoms were rated as either not present, suspected or definitely present (average-interrater-reliability: *κ* = 0.72). Present symptoms were only included in the score if not attributable to sleep, fever or substance use. PLIKS cases and controls were defined through presence or absence of definite or suspected PLIKS symptoms respectively.

### 2.4 Measurement of potential confounders

During the antenatal period information on potential confounders was obtained: maternal parity (0, 1, 2, or 3+) and age at birth of the study child, maternal cigarette smoking (‘No’, ‘Yes’) and alcohol consumption (‘Never’, ‘less than 1 glass per week’, ‘more than 1 glass per week’) during the first three months of pregnancy, maternal family history of depression (mother reporting on herself or her parents as suffering from depression; ‘No’, ‘Yes’); maternal education (‘Below O-level’, ‘O-level’ and ‘Above O-level’; O-levels are UK school-leaving qualifications taken at age 16), occupational social class (lower of either maternal or paternal social class; ‘Non-Manual’ and ‘Manual’; Dale and Marsh, 1993) and housing tenure (‘Mortgaged or owned’, ‘Privately rented’, ‘Council or Housing authority or Other’).

### 2.5 Genotyping

DNA was extracted as previously described (Jones et al., 2000). Genotyping of maternal and child DNA was performed by KBioscience Ltd. (www.kbioscience.co.uk) using a competitive allele-specific PCR system (KASPar). Genotypes in mothers (Mothers of cases: *p* = 0.11; mothers of controls: *p* = 0.86) and children (cases: *p* = 0.49; controls: *p* = 0.63) were in adherence to Hardy–Weinberg equilibrium, reaching a call rate of 95.2% and 93.8% respectively.

### 2.6 High-risk folate deficiency score

Folate-related nutritional and genetic information during early fetal life were also integrated into a high-risk folate deficiency score. For each single risk factor of folate deficiency present (i.e. no maternal folate supplementation at either 18 or 32 weeks, or a maternal dietary folate intake of one standard deviation (SD) below average at 32 weeks, or a maternal or child *MTHFR* C677T TT genotype), the score was increased by one (range 0 to 5).

### 2.7 Statistical methods

Associations between PLIKS and determinants of perinatal folate-status were assessed with logistic regression models and adjusted for sex (crude models). Models involving maternal folate intake were additionally adjusted for potential confounders as described above (adjusted models). Effect moderation, including sex-specific effects, maternal–child genotype and maternal folate -genotype interactions, was assessed with likelihood ratio tests (LRTs). Adherence of genotypes to Hardy–Weinberg-equilibrium was investigated with exact *χ*^2^-tests (Wigginton et al., 2005). All analyses were performed with the R software (CRAN, 2008).

To account for the possibility of unequal genotyping drop-out, sensitivity analyses was performed by comparing PLIKS cases and controls according to their expected parental mating type thus allowing for non-random genotype distributions. As parental mating types cannot be unequivocally derived from mother–child dyad information, case and control-specific mating type frequencies were estimated from the observed mother–child dyad frequencies using a simple Bayesian model (WinBUGS; Lunn et al., 2000; for details see Online supplement). Estimated mating type counts in cases and controls were formally compared using *χ*^2^-tests (see Online supplement Table 2).

## 3 Results

### 3.1 Sample selection

#### 3.1.1 Nutrient sample

Among 13,988 eligible singleton one-year-survivors and 13617 eligible mothers, who gave birth to a one-year surviving singleton child, there were 5344 mother–child pairs (38.2% eligible children; 39.2% eligible mothers) of White European origin with information on maternal folate intake and potential confounders during pregnancy, and PLIKS in children.

#### 3.1.2 Child *MTHFR* C677T sample

*MTHFR* C677T genotypes were available in 8645 individuals (61.8% eligible children). 4780 (34.17%) of these had data on PLIKS and were of White origin.

#### 3.1.3 Maternal *MTHFR* C677T sample

*MTHFR* C677T was successfully genotyped in 8069 (59.3%) eligible mothers. 4109 (30.2%) of these had children, who were assessed for the presence of PLIKS and 3943 of those mother–child pairs were of White origin (29.0% eligible mothers; 28% eligible children).

#### 3.1.4 Nutrient–genotype interaction sample

2845 mother–child pairs (20.9% eligible mothers, 20.3% eligible children) had complete information on maternal and child *MTHFR* C677T genotypes, maternal folate intake during pregnancy, PLIKS in children and potential confounders. Characteristics for each sample are given in Table 1.

The prevalence of ALSPAC children with definite/suspected PLIKS within this study was approximately 10 to 11% (see Table 1). This is lower than previously reported figures on the same cohort (Horwood et al., 2008).

### 3.2 Association between maternal folate intake during pregnancy and PLIKS

The relationship between maternal folate intake during pregnancy and PLIKS in children was investigated using the most complete phenotypic information available (nutrient sample, see Table 2).

There was no evidence that an overall high-risk folate deficiency score during pregnancy was related to PLIKS (Nutrient sample; 0.13 < *p* ≤ 0.59, adjusted model, see Table 2). As the effects of folate deficiency however may depend on the embryogenetic phase (Dolinoy et al., 2007), we also examined the effects of nutritional folate intake at 32 weeks and folate supplementation at 18 and 32 weeks. We observed that maternal folate supplementation at 18 weeks nominally increased the odds for PLIKS in children (OR = 1.34; 95%CI: [1.00;1.76]; *p* = 0.047, adjusted model), but found no effects for folate supplementation and dietary folate intake during later pregnancy.

### 3.3 Association between *MTHFR* C677T and PLIKS

The association between PLIKS and *MTHFR* C677T in mothers and children respectively was studied by exploiting all available genetic information (Child and maternal *MTHFR* C677T sample, see Table 3). A recessive genetic model was assumed as this model showed superior model fitting in particular for maternal genotype effects (Recessive: Akaike information criterion (AIC) = 2595; log-additive: AIC = 2598), although this was less pronounced for child genotype effects (Recessive: AIC = 3310; log-additive: AIC = 3310). Genetic association analysis identified a trend for an association between maternal *MTHFR* C667T and PLIKS such as that children of homozygous TT mothers showed 0.72 lower odds (95%-CI: [0.50;1.02]) of expressing these symptoms than children of CC/TC mothers. This effect appeared to be sex-specific (*p* = 0.029) and was observed in boys only (OR = 0.44, 95%-CI: [0.22;0.79]). There was no evidence for a relationship between child genotype and PLIKS, or sex-specific child genotype effects (*p* = 0.62). There was also no support for a maternal or child *MTHFR* C667 genotype effect when assuming an underlying log-additive genetic disease model (data not shown).

### 3.4 Effect moderation of folate-related risk factors for schizophrenia and PLIKS

Interactions between genetic and environmental determinants of fetal folate-status were explored using the smaller nutrient–genotype interaction sample (see online Supplement Table 1). The main effects for *MTHFR* C677T and maternal folate intake in this restricted sample were consistent with those reported above, although the confidence intervals were wider, and none of the results approached significance (see online Supplement Table 1).

Maternal folate intake effects did not vary with regard to genetic *MTHFR* background (interaction with maternal genotype: 0.34 < *p* ≤ 0.37; interaction with child genotype: 0.060 < *p* < 0.56, adjusted model). A trend for an interaction was observed between folate supplementation at 32 weeks and child *MTHFR* C677T (*p* = 0.060, adjusted model).

There was no evidence for a trans-generational *MTHFR* mother–child interaction (*p* = 0.46; crude model, data not shown) and no support was found for a combined effect of genetic and dietary/supplementary risk factors of folate deficiency (High-risk folate deficiency score: OR = 0.95, 95%CI: [0.78;1.15]).

### 3.5 Adjustment for multiple testing

Assuming at least three independent tests (for maternal/child *MTHFR* C677T genotype, maternal folate supplementation at 18 and 32- weeks gestation, and maternal dietary folate intake at 32- weeks gestation), there was no evidence for an overall association between determinants of fetal folate status and PLIKS in children at age 12 after Bonferroni-correction, or for overall interaction effects including sex-specific effects (although the number of performed interactions tests was much higher).

### 3.6 Population genotype distribution of *MTHFR* C677T

*MTHFR* C677T genotype distributions might be distorted as a result of unequal sample drop-out or random fluctuation due to small sample numbers. Mating type-frequencies were estimated for PLIKS cases and controls separately using a Bayes approach (gene-nutrient interaction sample, see Online Supplement) but showed only marginal deviation from Hardy–Weinberg expected frequencies (∼ 2%) for some mating type probabilities (see Online Supplement Table 2). There was no evidence for differences in mating type frequencies when comparing estimated counts between cases and controls (*χ*^2^(5) = 1.16, *p* = 0.94).

## 4 Discussion

This study investigated the association between markers of folate-status *in utero* and risk of developing psychotic symptoms at age 12. Our analyses focused on the direct and moderating influences of maternal folate supplementation assessed at 18 and 32- weeks gestation, maternal dietary folate intake at 32- weeks gestation and maternal and child *MTHFR* C667T genotypes.

Overall, we found no support for the hypothesis that determinants of impaired perinatal folate-status increase risk of non-clinical psychotic symptoms, as is commonly hypothesised for schizophrenia. There was nominal evidence for higher odds of PLIKS in children when mothers supplemented their diet with folate early during pregnancy (18 weeks). These results were consistent in their direction of association with the observed maternal *MTHFR* C677T effect (recessive model only); in particular boys had lower odds of expressing PLIKS when their mothers carried the TT genotype, which is associated with lower folate-status (Brattström et al., 1998; Friso et al., 2002). However, there was no evidence that the studied risk factors acted jointly or through gene-nutrient interactions, and none of the reported overall findings was significant when adjusted for multiple testing. Although observed associations for folate supplements at 18 weeks and maternal TT genotypes may suggest risk effects of higher fetal folate-status, especially in boys, they are likely to represent chance findings.

The power of our study to detect genetic and environmental effects was good to excellent. For example, within the Child *MTHFR* C667T sample the power approached 75% (recessive model) to approximately 100% (log-additive model) to detect a moderate recessive (OR = 1.44)(Gilbody et al., 2007) or log-additive genotype-risk effect respectively assuming a *MTHFR* 677T allele frequency of 0.33 and a PLIKS population risk of 10% (Gauderman and Morrison, 2006). Likewise, the power to detect a protective folate supplement effect (OR = 0.70) within the nutrient sample, assuming for example a 24% prevalence of maternal folate supplementation, as observed at 32 weeks, was high (91%). In view of the inherent study power, it is therefore unlikely that we have missed genetic or dietary/supplement effects that relate to fetal folate status during pregnancy, if they exist as hypothesised for schizophrenia. Their absence however might be related to several reasons: First, the relationship between internal folate-status and mental health outcomes at age 12 is likely to be complex. This may involve interactions between *MTHFR* C677T genotype and folate status (Brattström et al., 1998; Friso et al., 2002) but also between folate and other key determinants of the folate cycle such as those that are regulated by vitamin B12 (e.g. Selhub et al., 2009). However, our power to detect effect moderation was low. Assuming an alleviation of the genotype risk through folate supplementation (OR = 0.50) and otherwise similar conditions for a recessive genetic effect as outlined above, our interaction sample had only 25% power to detect gene–nutrient interactions (Gauderman and Morrison, 2006). Second, the effect of folate intake during pregnancy may depend on the embryogenetic phase as the epigenetic architecture of the fetal genome is established during time-sensitive periods (Dolinoy et al., 2007). It is therefore possible that the effect of fetal folate status on later mental health outcome varies during the course of the embryonic development and that our measures of maternal folate intake were inadequate at measuring the true exposure during these specific periods. Third, it is not clear to what extent non-clinical psychotic symptoms as assessed in this study reflect the same pathology underlying chronic and severe psychotic disorders such as schizophrenia. Although most established risk factors for schizophrenia also show associations with non-clinical psychotic experiences (Schreier et al., 2009; van Os et al., 2009; Zammit et al., 2009), findings across these phenotypes are not always consistent (Zammit et al., 2008). Our findings may therefore have relevance for studying the aetiology of non-clinical psychotic symptoms during childhood and adolescence, and contribute to the identification of risk factors that distinguish psychosis-like symptoms from schizophrenia. Fourth, we had no direct measure of folate exposure *in utero*. Although both, nutrition scores based on self-reported FFQs (Kleiser et al., 2009) and *MTHFR* genotypes (Frosst et al., 1995), have been related to serum/plasma homocysteine levels, misclassification will be present to some extent. This may have led to underestimating associations if misclassification is assumed to be non-differential with respect to psychotic symptoms, as seems most probable.

No evidence was found for bias due to non-random genotyping dropout when examining differences in estimated mating-type probabilities in PLIKS cases and controls. *MTHFR* C667T genotype distributions are furthermore unlikely to be confounded through socio-economic factors (Davey Smith and Ebrahim, 2003). Nonetheless, residual confounding remains a possibility that may have affected the selected measures of folate supplementation and dietary intake although our analyses were adjusted for a wide range of confounders including maternal parity, maternal cigarette smoking and alcohol consumption during the first three months of pregnancy, maternal family history of depression, maternal education, occupational social class, and housing tenure.

In conclusion we found no evidence that markers of folate status *in utero* were associated with risk of developing psychotic symptoms in adolescence.

## Role of the funding source

The UK Medical Research Council (grant number 74882), the Wellcome Trust (grant number: 076467/Z05/z) and the University of Bristol provided core support for ALSPAC and this work. SZ is funded through a Clinician Scientist Award funded by the National Assembly for Wales.

## Contributors

BG and SZ designed the study. BG, SL and SZ managed the literature searches. BG and AA performed the statistical analyses. BG, AA, SL, PE, GL, GDS and SZ contributed to the writing of the manuscript. This publication is the work of the authors and they will serve as guarantors for the contents of this paper.

## Conflict of interest

All other authors declare that they have no conflicts of interest.

## Figures and Tables

**Table 1:**
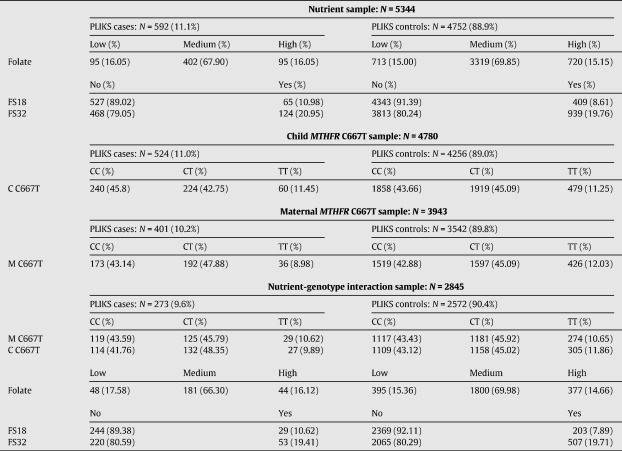
Sample descriptions.

M C667T — Maternal *MTHFR* C677T; C C677T — Child *MTHFR* C677T; FS18/FS32 — Folate supplementation at 18- and 32-week gestation; Folate — Maternal dietary folate intake at 32 weeks gestation; Low/Medium/High Folate — Low (intake ≤ mean − one SD), medium (mean − one SD < intake < mean + one SD ) or high (mean + one SD ≤ intake) (Categorisation for descriptive analysis only); SD — Standard deviation.

**Table 2:** Association between maternal folate intake during pregnancy and PLIKS.

	Unadjusted			Adjusted		
	OR [95% CI]	*P*	*P*_Sex_	OR [95% CI]	*P*	*P*_Sex_
*All (*N* = 5344, Cases = 592)*						
FS18^a^	1.31 [0.99;1.72]	0.063	0.37	1.34 [1.00;1.76]	0.047	0.38
FS32^a^	1.08 [0.87;1.33]	0.49	0.23	1.11 [0.90;1.38]	0.32	0.26
Folate^b^	0.99 [0.91;1.09]	0.89	0.13	1.03 [0.93;1.12]	0.60	0.19
Folate deficiency^c^	0.99 [0.81;1.20]	0.89	0.53	0.92 [0.75;1.12]	0.40	0.71

*Boys (*N* = 2613, Cases = 280)*						
FS18^a^	1.49 [1.00;2.17]	0.052	–	1.50 [1.00;2.19]	0.051	–
FS32^a^	1.22 [0.91;1.63]	0.18	–	1.22 [0.90;1.64]	0.19	–
Folate^b^	1.07 [0.94;1.22]	0.32	–	1.08 [0.95;1.24]	0.23	–
Folate deficiency^c^	0.92 [0.69;1.23]	0.58	–	0.90 [0.67;1.20]	0.48	–

*Girls (*N* = 2731, Cases = 312)*						
FS18^a^	1.15 [0.76;1.69]	0.48	–	1.19 [0.78;1.75]	0.40	–
FS32^a^	0.95 [0.69;1.27]	0.72	–	0.99 [0.72;1.35]	0.97	–
Folate^b^	0.93 [0.82;1.05]	0.25	–	0.97 [0.86;1.11]	0.69	–
Folate deficiency^c^	1.05 [0.80;1.37]	0.74	–	0.93 [0.71;1.22]	0.60	–

Models were adjusted for potential maternal confounders (parity, age at birth of the study child, cigarette smoking and alcohol consumption during the first three months of pregnancy, family history of depression, education), occupational social class, housing tenure and offspring sex (complete sample only). FS18/FS32 — Folate supplementation at 18 or 32- weeks gestation; OR — Odds ratio; Folate — Maternal dietary folate intake at 32 weeks gestation; Folate deficiency — High-risk folate deficiency score; *P*_Sex_ — Sex specific effect; SD — Standard deviation.

a ORs are given for presence vs. absence of folate supplementation.

b ORs are given for an increase in one SD of dietary folate intake.

c ORs are given for an increase in one score unit.

**Table 3:** Association between *MTHFR* C677T and PLIKS.

Genotype	Sample	*N*	Cases	OR [95% CI]^a^	*P*	*P*_Sex_
M C677T	All	3943	401	0.72 [0.50;1.02]	0.062	0.029
	Boys	1950	194	0.44 [0.22;0.79]	0.0041	–
	Girls	1993	207	1.00 [0.63;1.53]	0.99	–
C C677T	All	4780	524	1.02 [0.76;1.35]	0.89	0.62
	Boys	2396	253	1.10 [0.72;1.62]	0.66	–
	Girls	2384	271	0.95 [0.62;1.40]	0.80	–

Models were adjusted for offspring sex (complete sample only). M C667T — Maternal *MTHFR* C677T; C C677T — Child *MTHFR* C677T; P_Sex_ — Sex specific effect; OR — Odds ratio.

a ORs are given for TT vs CC/TC genotypes.
