# Supplementary material for: Perinatal folate-related exposures and risk of psychotic symptoms in the ALSPAC birth cohort
Source: Schizophr Res. 2010 Jul;120(1-3):177–83. doi: 10.1016/j.schres.2010.03.006 (PMC2927900; doi:10.1016/j.schres.2010.03.006)
Supplement: Supplementary file 1 — Online Supplement [file mmc1.doc]

**Online** **Supplement**

Online supplement 1: Estimation of mating type frequencies

Mating type frequencies in cases versus controls (see Online supplement Table 2) were estimated from a sample of 2845 individuals with complete genetic and phenotypic information (interaction sample). They were derived from mother-child dyad distributions according to population genetic principles (Schaid & Sommer, 1993; Shi et al., 2008) using a simple Bayesian model. The estimation of mating type probabilities was facilitated using weak Dirichlet priors and conditional allele transmission probabilities given the parental mating type. These priors were based on the expected distribution of genotypes according to Hardy-Weinberg equilibrium. The model assumed parental mating symmetry and exchangeability of parental genotypes (Shi et al., 2008) but allowed for non-random transmission of alleles (see below). Estimated mating type frequencies can be found in Online supplement Table 1.

The multinomial random variable y_i_, with 7 different outcomes representing the dyad types, and sample size n_i_, the total number of observed dyads, is distributed as y_i_ ~ Multin (n_i_; d_iM2C2_, d_iM1C2_, d_iM2C1_, d_iM1C1_, d_iM0C1_, d_iM1C0_, d_iM0C0_)

where d_ij_ represents the probability of the jth category dyad types:

d_M2C2_ = mt_P2P2_ + 0.5*t_C2|P2P1_*mt_P2P1_

d_M1C2_ = 0.5*t_C2|P2P1_*mt_P2P1_ + t_C2|P1P1_*mt_P1P1_

d_M2C1_ = 0.5*t_C1|P2P1_*mt_P2P1_ + 0.5*mt_P2P0_

d_M1C1_ = 0.5*t_C1|P2P1_*mt_P2P1_ + t_C1|P1P1_*mt_P1P1_ + 0.5*t_C1|P1P0_*mt_P1P0_

d_M0C1_ = 0.5mt_P2P0_ + 0.5*t_C1|P1P0_*mt_P1P0_

d_M1C0_ = t_C0|P1P1_*mt_P1P1_ + 0.5*t_C0|P1P0_*mt_P1P0_

d_M0C0_ = 0.5*t_C0|P1P0_*mt_P1P0_ + mt_P0P0_

d – Mother – Child dyad probability; mt – Mating type probability; M – Mother; C – Child; P – Parent; 0,1,2 – *MTHFR* C677T genotypes are given as counts of the *MTHFR* T allele; t – conditional probability for an allele being transmitted given the parental mating type

In accordance with the analysis of genotype distributions, none of the observed dyad frequencies showed detectable deviations from Hardy-Weinberg equilibrium (p_Cases_ = 0.49, p_Controls_ = 0.97).

##### Supplement references

Hartl, D.L., Clark, A.G., (1997). Principles of Population Genetics (Vol. 3). third ed. Sunderland: Sinauer Associates, Inc, Canada.

Schaid, D.J., Sommer, S.S., (1993). Genotype relative risks: methods for design and analysis of candidate-gene association studies. Am. J. Hum. Genet. 53(5) 1114-26.

Shi, M., Umbach, D.M., Vermeulen, S.H., Weinberg, C.R., (2008). Making the most of case-mother/control-mother studies. Am. J. Epidemiol. 168(5) 541-547.

Online supplement Table 1: Odds ratios for PLIKS (N=2845,Cases=273)

|  | Unadjusted | | | |
| --- | --- | --- | --- | --- |
|  | OR [95% CI] | P | P_M C677T_ | P_C C677T_ |
| M C677T ^a^ | 0.82[0.53;1.22] | 0.33 | - | - |
| C C677T ^a^ | 1.00[0.65;1.47] | 0.99 | - | - |
| FS18 ^b^ | 1.39[0.90;2.06] | 0.13 | 0.30 | 0.33 |
| FS32^b^ | 0.98[0.71;1.33] | 0.89 | 0.39 | 0.056 |
| Folate^c^ | 0.97[0.85;1.11] | 0.69 | 0.32 | 0.52 |
| Folate deficiency^d^ | 0.97[0.80;1.17] | 0.76 | - | - |
|  | Adjusted | | | |
|  | OR [95% CI] | P | P_MatC677T_ | P_ChC677T_ |
| M C677T ^a^ | 0.81[0.52;1.21] | 0.33 | - | - |
| C C677T ^a^ | 0.98[0.64;1.45] | 0.99 | - | - |
| FS18 ^b^ | 1.39[0.90;2.07] | 0.13 | 0.34 | 0.36 |
| FS32 ^b^ | 0.98[0.70;1.33] | 0.88 | 0.36 | 0.060 |
| Folate^c^ | 0.99[0.86;1.14] | 0.90 | 0.37 | 0.56 |
| Folate deficiency^d^ | 0.95[0.78;1.15] | 0.59 | - | - |

Models were adjusted for potential maternal confounders (parity, age at birth of the study child, cigarette smoking and alcohol consumption during the first three months of pregnancy, family history of depression, education), occupational social class, housing tenure and offspring sex(complete sample only).

M C667T – Maternal *MTHFR* C677T; C C677T – Child *MTHFR* C677T ; FS18/FS32 - Folate supplementation at 18- or 32-weeks gestation; Folate – Maternal dietary folate intake at 32 weeks gestation; Folate deficiency – High-risk folate deficiency score; P_M_ *_MTHFR_* _C667T_ / P_C_ *_MTHFR_* _C667T_ – Folate x genotype interaction effect; OR – Odds ratio; SD – Standard deviation

a – ORs are given for TT vs CC/TC genotypes

b – OR are given for presence vs. absence of folate supplementation

c – OR are given for an increase in 1 SD of dietary folate intake

d – OR are given for an increase in 1 score unit

Online Supplement Table 2: Mother-child dyad and mating type frequencies at the *MTHFR* C677T locus (N = 2845)

| **Sample** | **MAF (%)** |  | Dyad frequencies (%) | | |  | **Mating type frequencies (%)** | | |
| --- | --- | --- | --- | --- | --- | --- | --- | --- | --- |
|  | **T-allele** | **Dyad (M/C)** | **Observed** | **HWE-Predicted** | **Bayes Predicted*** |  | **Mating Type(M/P)** | **HWE-Predicted** | **Bayes Predicted*** |
| **Controls** | 33.75 | TT/TT | 4.06 | 3.84 | 4.06[3.37;4.84] |  | TT/TT | 1.30 | 0.98[0.04;3.48] |
| N=2572 |  | TC/TT | 6.82 | 7.55 | 6.80[5.9;7.79] |  | TT/CT | 10.19 | 10.56[5.83;15.94] |
|  |  | TT/TC | 7.78 | 7.55 | 7.76[6.81;8.78] |  | TT/CC | 10.00 | 10.65[5.87;15.14] |
|  |  | TC/TC | 23.20 | 22.36 | 23.15[21.61;24.73] |  | TC/TC | 20.00 | 20.05[14.53;25.83] |
|  |  | CC/TC | 14.75 | 14.81 | 14.78[13.49;16.13] |  | TC/CC | 39.25 | 40.16[30.24;48.82] |
|  |  | TC/CC | 15.50 | 14.81 | 15.43[14.13;16.81] |  | CC/CC | 19.26 | 17.30[12.82;22.92] |
|  |  | CC/CC | 27.89 | 29.08 | 27.96[26.33;29.63] |  |  |  |  |
|  |  |  |  |  |  |  |  |  |  |
| **Cases** | 33.28 | TT/TT | 4.14 | 3.68 | 4.18[2.45;6.35] |  | TT/TT | 1.23 | 0.89[0.04;3.47] |
| N=273 |  | TC/TT | 6.21 | 7.39 | 6.08[3.94;8.97] |  | TT/CT | 9.83 | 9.54[5.12;15.09] |
|  |  | TT/TC | 5.86 | 7.39 | 6.19[4.13;8.75] |  | TT/CC | 9.86 | 8.96[4.78;13.85] |
|  |  | TC/TC | 24.14 | 22.20 | 23.46[19.34;27.95] |  | TC/TC | 19.72 | 21.10[14.35;28.45] |
|  |  | CC/TC | 15.86 | 14.81 | 15.96[12.34;19.91] |  | TC/CC | 39.54 | 41.32[31.26;51.41] |
|  |  | TC/CC | 17.93 | 14.81 | 16.93[13.23;21.12] |  | CC/CC | 19.82 | 17.56[11.77;23.96] |
|  |  | CC/CC | 25.86 | 29.71 | 26.80[22.31;31.49] |  |  |  |  |
|  |  |  |  |  |  |  |  |  |  |

MAF – Minor allele frequency of *MTHFR* 677T in children; HWE – Hardy-Weinberg equilibrium; Dyad – Mother-Child dyad; *Median estimates of the posterior density, the sum of all frequencies will be due to the estimation process marginally different from 1, 95%-Credible intervals are given in parentheses (50000 iterations); M – Maternal; P – Paternal; C – Child; Estimation is based on a sample with complete information on maternal and child *MTHFR* C677T genotypes, maternal folate intake during pregnancy, psychosis-like symptoms in children, and potential confounders (Interaction sample)
